# Pulmonary sequestration in adult patients: a single-center retrospective study

**DOI:** 10.1186/s12931-023-02320-w

**Published:** 2023-01-12

**Authors:** Siying Ren, Lulu Yang, Ying Xiao, Zhongyi Tong, Li Wang, Yan Hu

**Affiliations:** 1 Department of Respiratory and Critical Care Medicine, The Second Xiangya Hospital of Central South University, Changsha, China; 2 Research Unit of Respiratory Disease, Central South University, Changsha, China; 3 Hunan Diagnosis and Treatment Center of Respiratory Disease, Changsha, China; 4 Department of Thoracic Surgery, The Second Xiangya Hospital of Central South University, Changsha, China; 5 Hunan Key Laboratory of Early Diagnosis and Precision Treatment of Lung Cancer, The Second Xiangya Hospital of Central South University, Changsha, China; 6 Department of Pathology, The Second Xiangya Hospital of Central South University, Changsha, China

**Keywords:** Pulmonary sequestration, CT findings, Arterial supply, Minimal invasive surgery, Thoracotomy

## Abstract

**Background:**

Pulmonary sequestration (PS) is a rare congenital lower airway malformation. This study presents the clinical and imaging features and surgical outcomes of PS in adults, and compare the safety and feasibility of minimally invasive surgery versus open thoracotomy for PS.

**Methods:**

Adult patients with PS treated at our center from July 2011 to September 2021 were included. Information regarding the patient demographics, clinical and CT features, arterial supply and venous drainage, and surgical outcomes were collected.

**Results:**

Ninety seven patients were included. The most common CT findings were mass lesions (50.5%) and cystic lesions (20.6%). The vast majority of the lesions (96 out of 97) were located close to the spine in the lower lobes (left vs. right: 3.6 vs. 1). Arterial supply was mainly provided by the thoracic aorta (87.4%) and abdominal aorta (10.5%). Intralobar and extralobar PS accounted for 90.7% and 9.3% of the patients, respectively. Three (4.5%) patients who underwent minimally invasive surgery were converted to open thoracotomy due to dense adhesions. Though no significant differences regarding operative time (P = 0.133), the minimally invasive surgery group was significantly better than the open thoracotomy group regarding intraoperative blood loss (P = 0.001), drainage volume (P = 0.004), postoperative hospital days (P = 0.017) and duration of chest drainage (P = 0.001). There were no cases of perioperative mortality. Only four (4.1%) patients developed postoperative complications, and no significant difference existed between the two groups.

**Conclusion:**

Our study revealed PS can present with a variety of different clinical and radiologic manifestations. Clinicians should consider the possibility of PS when diagnosing a lesion in the lower lobes close to the spine. Moreover, minimally invasive surgery is a safe and effective treatment modality for the treatment of PS in an experienced center.

## Background

Pulmonary sequestration (PS) is a rare congenital lower airway malformation that presents as a nonfunctioning pulmonary mass that does not communicate with the tracheobronchial tree and does not receive arterial blood from the pulmonary artery but from the systemic circulation. PS is classified into intralobar and extralobar according to the presence or absence of a separate visceral pleura covering the lung parenchyma and venous drainage [1]. The pathogenesis and etiology of PS are controversial [2]. In the past, both intralobar and extralobar PS were considered to be the formation of accessory lung buds caudal to normal lung buds with the same embryogenic origin [3]. Intralobar PS is now more commonly thought of as an acquired disease associated with bronchial obstruction, pneumonia or pleuritis, and rarely associated with other congenital diseases [4]. On the contrary, extralobular PS has been thought to be a congenital disorder originating from the primitive foregut, often accompanied by other congenital anomalies such as congenital diaphragmatic hernia and congenital cystic adenomatoid malformation [5].

Considering that most of the current data related to PS are from the pediatric literature, there are few reports of large institutional case series on PS in adults [6]. This study retrospectively analyzed the demographic characteristics, symptoms, CT and intraoperative findings, and postoperative course of adult PS patients treated surgically at our center, with the aim of better understanding this disease.

## Methods

### Patient selection and data collection

This study was conducted at the Second Xiangya Hospital of Central South University, and has been approved by the Clinical Research Ethics Committee (LYF2021170). This study was conducted in accordance with the Declaration of Helsinki and written informed consent was obtained from all patients. Adult patients (age 18 or greater) who underwent surgical treatment for PS at our center between July 2011 and September 2021 were included in this study. Patients with unavailable clinicopathological data were excluded. The following information was collected for analysis: age, gender, BMI, smoking history, symptoms, CT data, preoperative diagnosis, arterial supply and venous drainage, surgical details, and complications. Surgical complications were assessed according to the Society of Thoracic Surgeons database criteria. The choice of surgical approach (minimal invasive surgery vs. open thoracotomy) was decided at the discretion of the treating surgeon.

### Follow-up

Follow-up was performed through outpatient visits or telephone calls. The final follow-up visit was set at May 2022.

### Surgical procedure

**Fig. 1:**
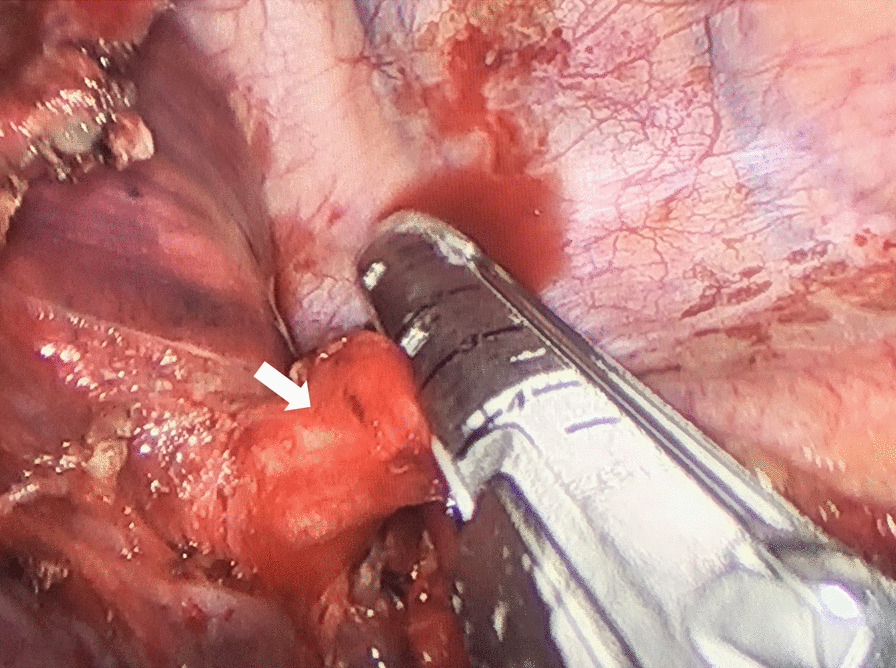
Cutting closure of the feeding artery (white arrow) using a stapling device under thoracoscopic approach

All patients underwent surgery in a lateral decubitus position with single lung ventilation on the contralateral side. After freeing the thoracic adhesions (if exist), meticulous intrathoracic exploration and dissection of the feeding artery from the aorta was performed initially. It was not necessary to completely free the adhesions around the feeding artery. To avoid retraction of the vascular stump proximal to the aorta, we tend to free the anomalous vessels on the side close to the lung tissue to leave enough space for cutting closure with the stapling device. Due to the high arterial pressure of the feeding artery, anesthesiologist was asked to perform controlled hypotensive measures (systolic blood pressure was controlled to below 90 mmHg) to prevent the nail compartment from splitting right before cutting closure of the feeding artery (see Fig. 1). Next, lobectomy or sublobar resection (including segmentectomy and wedge resection) is performed depending on the extent of the lung parenchymal lesion for intralobar PS patients and mass excision for extralobar PS patients.

### Perioperative antibiotic prophylaxis

For those complaining with cough or fever, regular anti-infective therapy was initially conducted before surgery. Surgery was then performed after infection was under control, demonstrated as recent absence of infective symptoms, no evidence of inflammation in CT scans, and normal blood routine examination. In addition, all patients received prophylactic anti-infective therapy during surgery and within 2 days after surgery.

### Statistical analyses

Normally distributed continuous variables were expressed as mean ± standard deviation; otherwise, they were expressed as median and range. Categorical variables were expressed as numbers and percentages. Normal distribution was assessed by Shapiro–Wilk *W* test. For comparisons of baseline characteristics between the two groups, independent samples *t*-test and Mann–Whitney test were used for normally and non-normally distributed continuous variables, respectively and Pearson’s chi-squared test for categorical variables. The data were analyzed using stata software. All statistical tests were two-sided and P < 0.05 was considered statistically significant.

## Results

### General characteristics

A total of 97 adult patients were included in this study, including 39 male and 58 female patients (male to female ratio of 0.67:1, Table 1). Patient age ranged from 18 to 65 years (median age 38 years). BMI ranged from 14.57 to 38.2 kg/m^2^ (median BMI 22.43 kg/m^2^). The majority of patients were nonsmokers (86/97, 88.7%). 75 (77.3%) patients complained of nonspecific symptoms. The most common symptoms were cough or sputum (58.7%), hemoptysis (28.9%) and fever (24.7%). 22 (22.7%) patients complained of incidental findings on CT scans performed for health examination. 59 (60.8%) patients underwent preoperative pulmonary function testing, of which 57 underwent pulmonary ventilation and diffusion function testing and 2 underwent pulmonary ventilation function testing only. Obstructive pulmonary ventilation dysfunction (OPVD) was found in 15 (25.4%) patients, all of whom were graded as mild OPVD. Restrictive pulmonary ventilation dysfunction (RPVD) was found in 4 (6.8%) patients, all of whom were graded as mild RPVD. There were 20 (35.1%) patients with pulmonary diffusion dysfunction (PDD), all of whom were graded as mild PDD.

**Table 1:** Demographic data and pulmonary function results for patients with pulmonary sequestration

Characteristics	Total	Open surgery	Minimal invasive surgery	P value
Age, years	38 (18–65)	39 (20–64)	36 (18–65)	0.325
Gender				0.674
Female	58(59.8%)	17(56.7%)	41 (61.2%)	
Male	39(40.2%)	13(43.3%)	26 38.8%)	
Smoking history				0.268
Current/former	11(11.3%	5(16.7%)	6 (9%)	
Never	86(88.7%)	25(83.3%)	61 (91%)	
BMI at presentation, kg/m2	22.43 (14.57–38.2)	21.64 (16.11–30.84)	22.68 (14.57–38.2)	0.109
Symptoms				0.673
Asymptomatic	22 (22.7%)	6 (20%)	16 (23.9%)	
Symptomatic	75 (77.3%)	24 (80%)	51 (76.1)	
Cough or expectoration	57 (58.7%)	22 (73.3%)	35 (52.2%)	
Hemoptysis	28 (28.9%)	9 (30%)	19 (28.4%)	
Fever	24 (24.7%)	8 (26.7%)	16 (23.9%)	
Chest/back pain	15 (15.5%)	5 (16.7%)	10 (14.9%)	
Shortness of breath	4 (4.1%)	1 (3.3%)	3 (4.5%)	
Chest distress	5 (5.2%)	1 (3.3%)	4 (6%)	
Pulmonary function^a^				
FEV1, percent predicted	95.39 ± 13.37	92.67 ± 14.06	96.08 ± 13.26	0.434
FVC, percent predicted	98.1 ± 12.3	96.17 ± 12.14	98.6 ± 12.42	0.546
FEV1/FVC, percent predicted	96.08 ± 9.39	94.08 ± 11.32	96.6 ± 8.9	0.413
DLCO, percent predicted	88 (63–198)	80.5 (63–100)	89 (63–198)	0.132
Mild OPVD	15(25.4%)	4 (33.3%)	11 (23.4%)	0.481
Mild RPVD	4 (6.8%)	1 (8.3%)	3 (6.4%)	0.81
Mild PDD	20 (35.1%)	6 (50%)	14 (31.1%)	0.223

Continuous variables were expressed as mean ± standard deviation or median and range, as appropriate. Categorical variables were expressed as numbers and percentages

OPVD, obstructive pulmonary ventilation dysfunction; RPVD, restrictive pulmonary ventilation dysfunction; PDD, pulmonary diffusion dysfunction

^a^Pulmonary function testing results available in 59 (60.8%) patients

### CT scans results

**Fig. 2:**
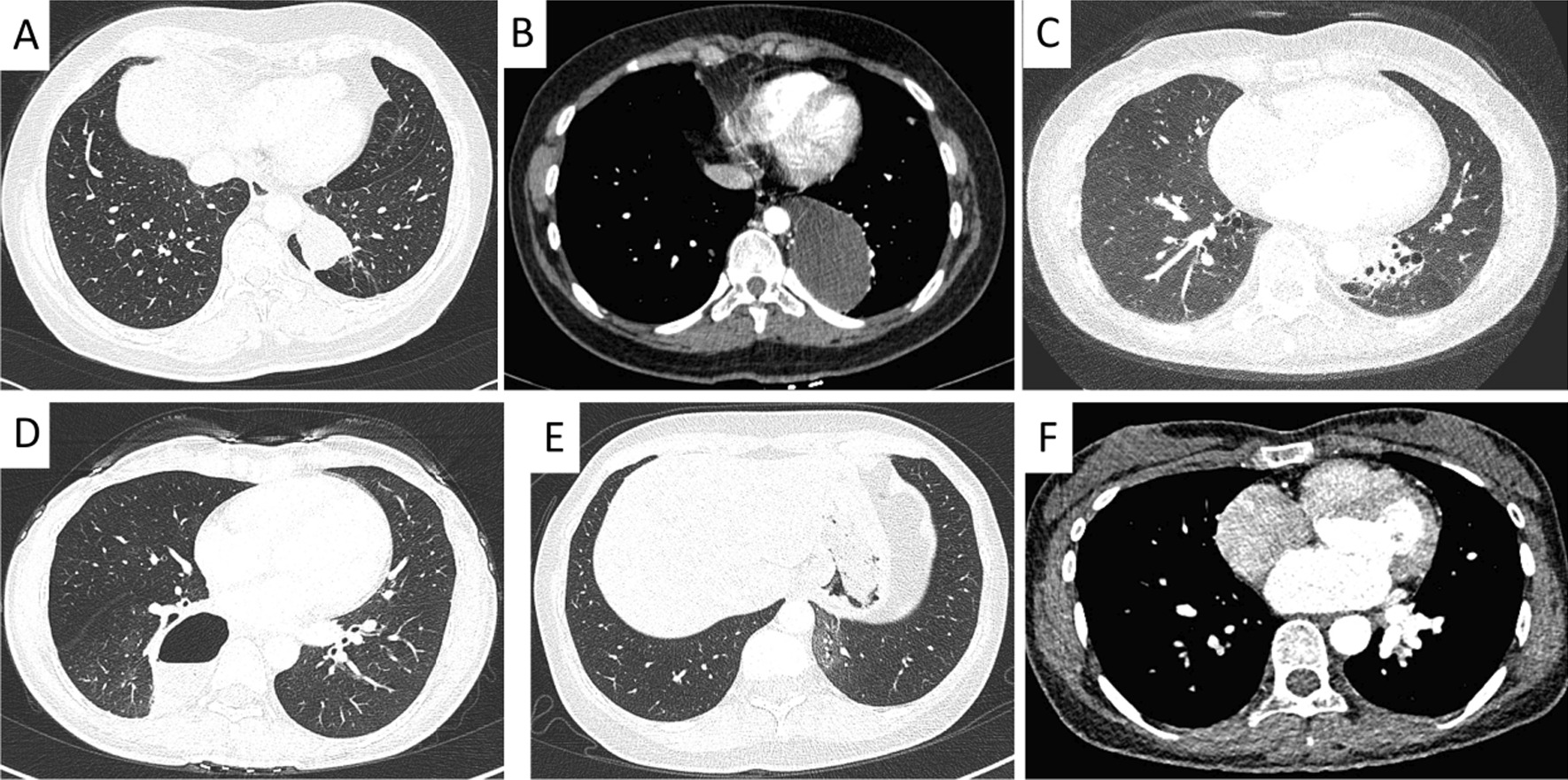
Representative CT appearances of pulmonary sequestration. **A** masse lesions; **B** cystic lesions; **C** bronchiectasis; **D** cavitary lesions; **E** pneumonic lesions; **F** intrapulmonary cord-like shadow

All patients underwent contrast-enhanced CT examination of the chest. Among them, 69 patients was immediately diagnosed with PS and the other 28 further underwent CT angiography to confirm the diagnosis of PS. Mass lesions (49/97, 50.5%, Table 2) were the most common CT presentation (see Fig. 2), followed by cystic lesions (20/97, 20.6%), cavitary lesions (10/97, 10.3%), bronchiectasis (6/97, 6.2%), pneumonic lesions (6/97, 6.2%) and intrapulmonary cord-like shadow (6/97, 6.2%). All lesions were located close to the spine in the lower lobes except for one (1%) extralobar lesion located in the left upper lobe. Of these, 75 lesions (77.3%) were located in the left lower lobe and 21 lesions (21.6%) were located in the right lower lobe.

**Table 2:** Chest CT presentation data for patients with pulmonary sequestration

Characteristics	Total	Open surgery	Minimal invasive surgery	P value
CT appearances				0.926
Mass lesion	49 (50.5%)	16 (53.3%)	33 (49.2%)	
Cystic lesion	20 (20.6%)	7 (23.3%)	13 (19.4%)	
Cavitary lesion	10 (10.3%)	2 (6.7%)	8 (11.9%)	
Pneumonic lesion	6 (6.2%)	2 (6.7%)	4 (6%)	
Bronchiectasis	6 (6.2%)	2 (6.7%)	4 (6%)	
Intrapulmonary cord-like shadow	6 (6.2%)	1 (3.3%)	5 (7.5%)	
Location				0.304
Left lower lobe	75 (77.3%)	22 (73.3%)	53 (79.1%)	
Right lower lobe	21 (21.6%)	7 (23.3%)	14 (20.9%)	
Left upper lobe	1 (1%)	1(3.3%)	0 (0%)	

The variables were expressed as numbers and percentages

### Arterial supply and venous drainage

The presence of supplying arteries from the systemic circulation was confirmed intraoperatively in all patients. Two (2.1%) cases of PS received the arterial supply from two different origins of artery (thoracic aorta and left internal thoracic artery in one case and celiac artery and intercostal artery in another case). The remaining 95 (97.9%) cases of PS had feeding artery of single origin, including 83 (87.4%) from the thoracic aorta, 10 (10.5%) from the abdominal aorta, 1 (1.1%) from the celiac trunk, and 1 (1.1%) from the left subclavian artery (Table 3). As for the number of supplying artery, there was only one supplying artery in 80 (82.5%) cases, two supplying arteries in 12 (12.4%) cases, and three supplying arteries in five (5.1%) cases. It was confirmed intraoperatively that the majority of cases refluxed to the pulmonary vein (94/97, 96.9%), two case (2.1%) to the azygous vein, and one case (1%) to the inferior vena cava.

**Table 3:** Data regarding arterial supply and venous drainage for patients with pulmonary sequestration

Characteristics	Total	Open surgery	Minimal invasive surgery	P value
Origin of feeding artery				0.339
2 origins	2 (2.1%)	0 (0%)	2 (3%)	
1 origin	95 (97.9%)	30 (100%)	65 (97%)	0.215
Thoracic aorta	83 (87.4%)	24 (80%)	59 (90.8%)	
Abdominal aorta	10 (10.5%)	5 (16.7%)	5 (7.7%)	
Celiac artery	1 (1.1%)	0 (0%)	1 (1.5%)	
Subclavian artery	1 (1.1%)	1 (3.3%)	0 (0%)	
Number of feeding artery				0.821
3	5 (5.1%)	2 (6.7%)	3 (4.5%)	
2	12 (12.4%)	3 (10%)	9 (13.4%)	
1	80 (82.5%)	25(83.3%)	55 (82.1%)	
Venous drainage				0.5
Pulmonary vein	94 (96.9%)	30 (100%)	64 (95.5%)	
Azygos vein	2 (2.1%)	0 (0%)	2 (3%)	
Inferior vena cava	1 (1%)	0 (0%)	1 (1.5%)	

The variables were expressed as numbers and percentages

### Surgical details

All patients underwent surgical treatment and were ultimately classified as 88 (90.7%) intralobar and 9 (9.3%) extralobar PS (Table 4). Minimally invasive surgery was performed in 67 (69.1%) patients, of which 60 (61.9%) patients underwent video-assisted thoracoscopic surgery (VATS) and 7 (7.2%) patients underwent robotic-assisted thoracoscopic surgery (RATS). Three (4.5%) patients was converted to thoracotomy for dense intrathoracic adhesions. 30 (30.9%) patients underwent open thoracotomy. For intralobar PS, 80 (90.9%) patients underwent lobectomy, two (2.3%) patients underwent segmentectomy, four (4.5%) patients underwent wedge resection, and two (2.3%) patients underwent supplying artery dissection only. All 9 extralobar PS patients underwent mass excision. Intrathoracic adhesions were present in 63 (64.9%) patients, of which 36 (37.1%) had extensive and dense intrathoracic adhesions.

**Table 4:** Surgical details and complications of patients with pulmonary sequestration

Characteristics	Total	Open surgery	Minimal invasive surgery	P value
Type of sequestration				0.553
Intralobar	88(90.7%)	28(93.3%)	60(89.6%)	
Extralobar	9(9.3%)	2(6.7%)	7(10.4%)	
Surgical approach				NA
Open thoracotomy	30(30.9%)			
RATS	7(7.2%)			
VATS	60(61.9%)			
Type of resection				0.649
Lobectomy	80(82.5%)	27(90%)	53(79.1%)	
Mass excision	9(9.3%)	2(6.7%)	7(10.4%)	
Wedge resection	4(4.1%)	1(3.3%)	3(4.5%)	
Segmentectomy	2(2.1%)	0(0%)	2(3%)	
Disconnection of aberrant feeding artery	2(2.1%)	0(0%)	2(3%)	
Intrathoracic adhesions	63(64.9%)	16(53.3%)	47(70.1%)	0.109
Dense intrathoracic adhesions	36(37.1%)	9(30%)	27(40.3%)	0.332
Conversion to open thoracotomy	3(4.5%)			NA
Operative time, min	120 (50–325)	115 (75–220)	120 (50–325)	0.133
Estimated blood loss, ml	70 (10–800)	92.5 (50–800)	60 (10–550)	0.001
Volume of drainage, ml	360 (40–2040)	430 (60–1630)	330 (40–2040)	0.004
Length of postoperative hospitalization, days	4 (1.5–10.8)	4.9 (2–10.8)	4 (1.5–8)	0.017
Chest tube duration, days	2.8 (0.8–7.1)	3.2 (1–7.1)	2.7 (0.8–6)	0.001
Postoperative complications	4(4.1%)	2(6.7%)	2(3%)	0.399
Atrial fibrillation	1(1%)	1(3.3%)	0(0%)	
Chylothorax	2(2.1%)	0(0%)	2(3%)	
Hemothorax required for reoperation	1(1%)	1(3.3%)	0(0%)	

Continuous variables were expressed as mean ± standard deviation or median and range, as appropriate. Categorical variables were expressed as numbers and percentages

NA, not available; RATS, robotic-assisted thoracoscopic surgery; VATS, video-assisted thoracoscopic surgery

The above-mentioned baseline characteristics were balanced between the two groups. Compared with open thoracotomy, minimally invasive surgery was significantly associated with less intraoperative blood loss (median, 60 vs. 92.5 ml, 0.001), less volume of drainage (median, 330 vs. 430 ml, P = 0.004), shorter postoperative hospital days (median, 4 vs. 4.9, P = 0.017) and shorter duration of chest drainage (median, 2.7 vs. 3.2, P = 0.001), though no significant differences existed in terms of operative time (median, 120 vs. 115 min, P = 0.133).

### Mortality and morbidity

No patient experienced perioperative death during hospitalization. Only four (4.1%) patients developed postoperative complications, and there was no significant difference between the minimal invasive surgery (2/67, 3%) and thoracotomy groups (2/30, 6.7%) in terms of complication rate (P = 0.399). A 62-year-old male patient who underwent lobectomy via thoracotomy developed atrial fibrillation on postoperative day 1. Sinus rhythm was restored after the administration of cordarone. A 41-year-old female patient developed chylothorax on postoperative day 2 after undergoing VATS lobectomy. Due to ineffective conservative treatment, heterotopic thoracic duct ligation under VATS was performed on postoperative day 4. She was discharged on 7 days after surgery. An extralobar PS patient developed chylothorax on postoperative day 2 after mass excision. He was recovered on a no-fat diet for 2 days and was discharged on postoperative 5 days. A 26-year-old male patient developed postoperative hemothorax after undergoing lobectomy via thoracotomy. He received an exploratory hemostasis under VATS at 7 days postoperatively due to the excessive postoperative drainage.

### Follow-up

Of the 97 patients, 30 (30.9%) were lost to follow-up. The follow-up time range was 4.5–125 months (median, 35 months), and all patients did not develop PS-related complications or relapse during follow-up.

## Discussion

PS is the second most common congenital lung malformation, accounting for 0.15–6.45% of all congenital lung malformations, with an incidence of about 1/20,000 in the population [7]. PS can be divided into intralobar and extralobar types depending on the presence or absence of a separate visceral pleura covering the lung parenchyma and venous drainage. Intralobar PS is commonly seen in the age group of 20 years and younger, but rarely diagnosed at age 50 or older [8]. A case series study from Mayo Clinic included 32 adults PS patients with a median age of 42 years (IQR 28–53) [6]. To the best of our knowledge, this is a single-center study with the largest sample size analyzing the clinical and imaging characteristics and surgical outcomes in adult PS to date. 97 adult PS patients were included in the study, with a median age of 38 years (interquartile range 29–48).

PS may present with cough, sputum, hemoptysis, fever, chest pain, and chest tightness, when accompanied with pulmonary infection [1]. PS can be asymptomatic and diagnosed incidentally when there is no pulmonary infection [9, 10]. In our study, 22.7% of patients did not complain of any specific symptoms and were found incidentally by CT scans of the chest during health examination.

Contrast-enhanced CT scans show that PS can present with a variety of different manifestations including masses, cysts, bronchiectasis or pulmonary atelectasis [3]. Sun et al. reported that the most common CT findings of PS were soft tissue shadow, cystic lesions, cavitary lesions and bronchiectasis [9]. Alsumrain et al. reported that masses/solid lesions (61% of cases) were the most common CT findings of PS [6]. Our study showed that the most common CT findings were mass lesions (50.5%) followed by cystic lesions (20.6%), cavitary lesions (10.3%), pneumonic lesion (6.2%), bronchiectasis (6.2%), and intrapulmonary cord-like shadow (6.2%).

The definite diagnosis of PS requires the identification of abnormal arterial supply to the sequestered lung tissues [11]. By searching the Chinese National Knowledge infrastructure (CNKI) database, Wei et al. found that the thoracic aorta (1384 cases, 76.55%) and the abdominal aorta (334 cases, 18.47%) were the two most common sources of arterial supply. The number of arterial supplies included one (79.09%), two (15.99%) and more than two (4.92%). Our findings were similar to their outcomes, showing that the arterial supply was mainly originated from the thoracic aorta (87.4%), followed by the abdominal aorta (10.5%). There were only one supplying artery in 80 (82.5%) cases, two in 12 (10.5%) and three in 5 (5.1%). It has been reported that the vast majority of venous drainage in intralobar PS was into the pulmonary veins [12]. For extralobar PS, however, it was drained into systemic veins, frequently inferior vena cava, azygos vein, or hemiazygos vein [13]. Our study showed that all of the PS veins reflowed to the pulmonary veins except for two (2.1%) vein that drained to the azygos vein (one for intralobar PS and one for extralobar PS) and one (1%) to the inferior vena cava (intralobar PS).

Although no established guidelines have been generated so far for the treatment of PS, it has been generally accepted that surgical resection is the preferred therapy modality for PS in most patients, regardless of the presence of symptoms [13, 14]. Surgical treatment can achieve the following objectives: removal of the lesion, definite confirmation of the diagnosis, avoidance of complication occurrence and controversial tumor formation [14, 15]. In recent years, VATS has gradually become the preferred treatment modality for PS due to the advantages of less postoperative pain, more aesthetics, and faster recovery [16]. The advantages and disadvantages of minimally invasive surgery versus open thoracotomy have been previously explored [17]. Wang et al. found that although there were no significant differences in operative time, postoperative hospital days, or complication rates, minimally invasive surgery had less intraoperative bleeding compared to open thoracotomy, but more postoperative drainage volume and longer duration of chest drainage. Liu et al. revealed that no significant differences were found between video-assisted thoracic surgery group and open thoracotomy group in terms of the duration of operation, blood loss, amount of chest drainage, duration of chest drainage, length of postoperative hospital stay, and complications [18]. Our results showed that there were no significant differences between the two groups in terms of operative time (P = 0.133). However, minimally invasive surgery was associated with less intraoperative blood loss (P = 0.001), less drainage volume (P = 0.004), shorter postoperative hospital days (P = 0.017) and shorter duration of chest drainage (P = 0.001), suggesting minimally invasive surgery as a safe and effective treatment modality in the treatment of PS.

Lobectomy is the recommended treatment for intralobar PS [16]. However, in terms of lung function preservation, sublobar resection is an alternative modality to lobectomy for peripheral lesions or small asymptomatic lesions [19]. In our study, segmentectomy and wedge resection was performed in two (2.1%) and four (4.1%) patients of intralobar PS, respectively. All of these patients complained complete remission and no recurrence during the follow-up visits (median follow-up period was 26 months). Dissection of the abnormal vessels only may be enough for intralobular PS with only hemoptysis and no symptoms of pulmonary infection [16]. Two patients in this study complained of hemoptysis only and preoperative CT scans showed intrapulmonary cord-like shadow, with no evidence of inflammation. They both underwent supplying artery dissection only, with complete remission and no recurrence during the follow-up visit (30 months and 19 months, respectively).

We acknowledge there are several limitations of this study needed be considered. First, this study involved a single center and was retrospective by design. Selection bias may have existed. Second, high follow-up lost rate (30 of 97, 30.9%) and relatively short follow-up period resulted in incomplete prognostic information of these patients.

## Conclusion

This study emphasizes the necessary awareness of treating clinicians that PS should be included in the differential diagnosis of lung lesion in the lower lobes close to the spine. Moreover, minimally invasive surgery is a safe and effective treatment modality for the treatment of PS in an experienced center.

## Data Availability

The datasets used and/or analysed during the current study are available from the corresponding author on reasonable request.

## Abbreviations

PS: Pulmonary sequestration
OPVD: Obstructive pulmonary ventilation dysfunction
RPVD: Restrictive pulmonary ventilation dysfunction
PDD: Pulmonary diffusion dysfunction
CNKI: Chinese National Knowledge Infrastructure
